# Eosinophil as a Protective Cell in *S. aureus* Ventilator-Associated Pneumonia

**DOI:** 10.1155/2013/152943

**Published:** 2013-09-03

**Authors:** Ana Rodriguez-Fernandez, David Andaluz-Ojeda, Raquel Almansa, Mar Justel, Jose Maria Eiros, Raul Ortiz de Lejarazu

**Affiliations:** ^1^Microbiology Service, Clinical University Hospital-SACYL, Avda Ramón y Cajal 3, 47005 Valladolid, Spain; ^2^Critical Care Medicine Service, Clinical University Hospital-SACYL, Avda Ramón y Cajal 3, 47005 Valladolid, Spain; ^3^Biomedical Investigation Unit, Clinical University Hospital (ibC), SACYL & IECSCYL, Avda Ramón y Cajal 3, 47005 Valladolid, Spain; ^4^National Centre of Influenza, Avda Ramón y Cajal 7, 47005 Valladolid, Spain

## Abstract

Cell counts of leukocytes subpopulations are demonstrating to have an important value in predicting outcome in severe infections. We evaluated here the render of leukogram counts to predict outcome in patients with ventilator-associated pneumonia (VAP) caused by *Staphylococcus aureus*. Data from patients admitted to the ICU of Hospital Clínico Universitario de Valladolid from 2006 to 2011 with diagnosis of VAP caused by *S. aureus* were retrospectively collected for the study (*n* = 44). Leukocyte counts were collected at ICU admission and also at VAP diagnosis. Our results showed that nonsurvivors had significant lower eosinophil counts at VAP diagnosis. Multivariate Cox regression analysis performed by the Wald test for forward selection showed that eosinophil increments from ICU admission to VAP diagnosis and total eosinophil counts at VAP diagnosis were protective factors against mortality in the first 28 days following diagnosis: (HR [CI 95%], *P*): (0.996 [0.993–0.999], 0.010); (0.370 [0.180–0.750], 0.006). Patients with eosinophil counts <30 cells/mm^3^ at diagnosis died earlier. Eosinophil counts identified survivors: (AUROC [CI 95%], *P*): (0.701 [0.519–0.882], 0.042). Eosinophil behaves as a protective cell in patients with VAP caused by *S. aureus*.

## 1. Introduction

*Staphylococcus aureus* has persisted as an important public health problem, mostly due to the emergence of strains that were resistant to methicillin and oxacillin (MRSA) in the 1960s [1]. Ventilator-associated pneumonia (VAP) is the most frequent infection among patients hospitalized in intensive care units (ICU), with *S. aureus* infection being a leading cause of VAP [2]. VAP maintains high morbidity and mortality. Because of that, a number of inflammatory biomarkers are under evaluation to guide duration of antibiotic therapy and to predict disease outcome in VAP, with heterogeneous results [3–5]. Cell counts of leukocytes subpopulations are demonstrating to have an important value in predicting outcome in severe infections [6–8]. Counting leukocytes is a routine and inexpensive test in ICU settings. We designed a retrospective study aimed to evaluate the influence of leukocyte subpopulations counts on the probability of death in patients with VAP caused by *S. aureus*. 

## 2. Methods

*Study Design and Subjects*. Data from patients admitted to the ICU of Hospital Clínico Universitario de Valladolid from 2006 to 2011 with diagnosis of VAP caused by *S. aureus* were retrospectively collected for the study (*n* = 44). Patients who were intubated and ventilated in the moment of ICU hospitalization and developed VAP were eligible. Patients who had been treated with corticosteroids or immunosuppressive drugs and immunocompromised patients were not eligible and were excluded from the present study. VAP was defined as the pneumonia arising more than 72 h after endotracheal intubation characterized by the presence of new or progressive radiographic infiltrate associated with two or more of the following criteria: (a) temperature of greater than 38.5°C or less than 36.5°C, (b) leukocyte count of greater than 12,000/*μ*L or less than 4,500/*μ*L, (c) purulent endotracheal aspirate, and (d) positive (≥10^6^ cfu/mL) endotracheal aspirate. Microbiological diagnosis was performed by quantitative cultures of lower respiratory tract samples (endotracheal aspirate (BAS) or bronchoscopic alveolar lavage (BAL)), following the standard protocols for diagnosis of respiratory bacteria employed in our center. Patients with positive microbiological identification before 72 first hours following mechanical ventilation instauration were excluded because they did not satisfy VAP definition. A standard survey was employed to collect clinical data and leukogram counts from the patients. Patient identification remained anonymous and informed consent was waived due to the observational nature of the study. Approval of the study protocol in both the scientific and the ethical aspects was obtained from the Scientific Committee for Clinical Research of our hospital. 

*Statistical Analysis. *For the demographic and clinical characteristics of the patients, differences between groups were assessed using the Chi-squared test for categorical variables and the Mann-Whitney *U* test for continuous variables when appropriate. We determined the hazard ratio (HR) and 95% confidence interval by Cox regression analysis, which was used to assess the impact of eosinophil increments and counts on mortality over time. Multivariate Cox regression analysis was performed by using the Wald test for forward selection. Statistical analysis was performed by using IBM-SPSS Statistics 20.0. 

## 3. Results

The vast majority of our patients were elderly males, being hypertension, cardiovascular disease, and smoker habit the most frequent comorbidities. Both survivors and nonsurvivors spent four days under mechanical ventilation and presented an APACHE-II score of 18 on average. They were comparable in terms of age, sex, and accompanying comorbidities. We defined coinfection as those other bacteria infecting any localization of our patients with clinical significance. Bacterial coinfection was principally of the following foci: respiratory, urine, and blood. The principles and most frequent pathogens isolated were Gram negative rods from Enterobacteriaceae family (43.3%), *Acinetobacter baumannii* (26.7%), and coagulase negative staphylococci (23.3%). 

**Figure 1:**
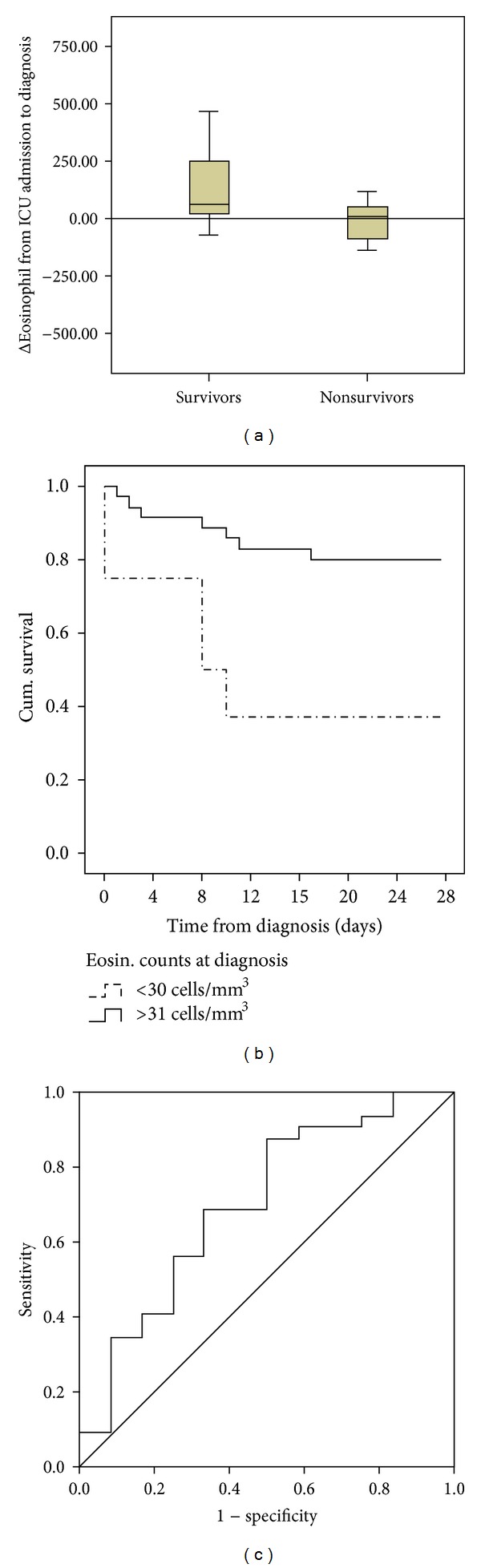
(a) Box plots showing increments of eosinophil counts: [counts at VAP diagnosis]–[counts at ICU admission] (*P* = 0.016). (b) Kaplan Meier curves for survival: deciles from percentile 10 to percentile 90 of eosinophil counts were calculated and used to compare survival times in those patients with low or high counts. The first decile showing significant differences between groups based upon the log-rank test was used as the cutoff (percentile 20). Time was censored at 28 days following VAP diagnosis. Cum. survival: cumulative survival. (c) AUROC analysis: the accuracy and the predictive values of eosinophil counts for detecting survivors in the first 28 days following VAP diagnosis were assessed calculating the AUROC.

The comparison of cell counts revealed significant lower eosinophil counts at VAP diagnosis in nonsurvivors (see Table 1). When cell increments ([counts at VAP diagnosis]–[counts at ICU admission]) were evaluated in survivors and nonsurvivors, nonsurvivors showed significant lower increments of eosinophil counts (*P* = 0.010) (see Figure 1(a)). Potential confounding variables introduced in the multivariate Cox regression analysis were age, sex, APACHE-II score, VAP caused by *Staphylococcus aureus* methicillin resistance/*Staphylococcus aureus *methicillin susceptible (MRSA/MSSA), bacterial coinfection, and days in mechanical ventilation. For eosinophil increments, the Wald test selected the APACHE-II score and the eosinophil increments as the variables associated with mortality: (HR [CI 95%], *P*): APACHE-II score: (1.073 [1.001–1.151], 0.050); (eosinophil increments): (0.996 [0.993–0.999], 0.010). For total eosinophil counts at VAP diagnosis (log values), the Wald test selected total eosinophil counts at VAP diagnosis (log values) as the only variable associated with prognosis: (HR [CI 95%], *P*): (0.370 [0.180–0.750], 0.006). Therefore, multivariate Cox regression analysis showed that eosinophil increments as well as total eosinophil counts at VAP diagnosis (log values) were protective factors against mortality in the first 28 days following diagnosis of VAP. Kaplan Meier analysis showed that patients with eosinophil counts less than 30 cells/mm^3^ died earlier (see Figure 1(b)). Area under the receiver operating characteristic curve (AUROC) analysis confirmed eosinophil counts at VAP diagnosis as a good test to diagnose survival (see Figure 1(c)): (area [CI 95%], *P*): (0.701 [0.519–0.882], 0.042). 

## 4. Discussion

Regression studies and AUROC analysis supported the protective role of eosinophils in VAP caused by *S. aureus*. Eosinophils are granulocytes that develop in the bone marrow from pluripotent progenitors. They are released into the peripheral blood in a phenotypically mature state, and they are capable of being activated and recruited into tissues in response to appropriate stimuli, most notably the cytokine interleukin-5 (IL-5) and the eotaxin chemokines [9]. Eosinophils are recruited to and activated in lung tissue as part of the pathophysiology of asthma, but recent findings confirm antimicrobial activities of eosinophils [9]. Catapult-like release of structures resembling neutrophil extracellular traps (NETs) from eosinophils has been documented [10]. Our results could in fact support the existence of an antibacterial activity of the eosinophil in the severe infection caused by *S. aureus*. In our patients, fail in expanding eosinophil counts was translated into a poorer outcome. 

There is increasing evidence on eosinophils as a protective cell in critically ill patients. Abidi et al. described eosinopenia as a marker of sepsis on admission to ICU [11]. Recently, these authors have described eosinopenia as an early marker of increased mortality in critically ill medical patients [12]. Merino et al. have reported lower eosinophil counts in patients who died of sepsis than in those who survived [6]. Prince et al. have demonstrated that *S. aureusα*-hemolysin induces cell death in eosinophils [13], which could represent a microbial mechanism of evasion from host immune response.

Terradas et al. observed that both sustained eosinopenia and a high neutrophil to lymphocyte count ratio were independent markers of mortality in patients with bacteremia [8]. Based upon these results, we evaluated the neutrophil to lymphocyte count ratio in survivors and nonsurvivors. No differences were found between groups for this ratio although there was a trend to exhibit a higher ratio in nonsurvivors (see Table 1).

A limitation of our study was that only two samples were collected (at admission and at VAP diagnosis). In further studies, it will be interesting to assess eosinophil counts in other extra time points during the disease course.

## 5. Conclusion

We document here for the first time a protective effect of eosinophils in patients suffering from VAP caused by *S. aureus*. Eosinophil counting is an inexpensive biomarker easy to implement in clinical practice. 

## Figures and Tables

**Table 1:** Clinical characteristics of survivors and nonsurvivors.

	Survivors (*n* = 26)	Nonsurvivors (*n* = 18)	*P *
Age (years)	60.0 (35.0)	63.5 (26.0)	n.s
Sex (male)	23 (88.5)	7 (38.9)	n.s
APACHE-II score	17.0 (11.0)	22.0 (12.0)	n.s
Days under mechanical ventilation until VAP diagnosis	4.0 (4.0)	4.5 (4.0)	n.s
MRSA/MSSA	8 (30.8)	4 (22.2)	n.s
Bacterial coinfection (Y/N)	20 (76.9)	10 (55.6)	n.s
Diabetes (type I or II) (Y/N)	4 (15.4)	2 (11.1)	n.s
Cardiovascular disease (Y/N)	6 (23.1)	5 (27.8)	n.s
Chronic renal disease (Y/N)	2 (7.7)	1 (5.6)	n.s
Chronic respiratory disease (Y/N)	6 (23.1)	0 (0.0)	n.s
Cerebrovascular disease (Y/N)	6 (23.1)	0 (0.0)	n.s
Smoker (ever) (Y/N)	8 (30.8)	1 (5.6)	n.s
Neurological disease (Y/N)	4 (15.4)	0 (0.0)	n.s
Hypertension (Y/N)	12 (46.2)	8 (44.4)	0.084
Hematologic malignancy (ever) (Y/N)	1 (3.8)	0 (0.0)	n.s
Cirrhosis of the liver (Y/N)	2 (7.7)	0 (0.0)	n.s
Metastatic solid cancer (ever) (Y/N)	0 (0.0)	2 (11.1)	0.018
Gastrointestinal disease (Y/N)	9 (34.6)	1 (5.6)	n.s
Chemotherapy (ever) (Y/N)	1 (3.8)	2 (11.1)	n.s
Alchohol abuse (Y/N)	4 (15.4)	0 (0.0)	n.s
Intravenous drug abuse (Y/N)	2 (7.7)	0 (0.0)	n.s
Obesity (Y/N)	3 (11.5)	1 (5.6)	n.s
Dyslipidemia (Y/N)	4 (15.4)	2 (11.1)	n.s
Lymphocytes at admision to ICU (cells/mm^3^)	960.9 (1017.3)	812.3 (1336.9)	n.s
Monocytes at admision to ICU (cells/mm^3^)	480.3 (442.9)	465.8 (436.6)	n.s
Neutrophils at admision to ICU (cells/mm^3^)	7801.9 (7053.9)	9504.0 (9101.8)	n.s
Basophils at admision to ICU (cells/mm^3^)	11.5 (22.0)	10.5 (28.0)	n.s
Eosinophils at admision to ICU (cells/mm^3^)	14.9 (119.0)	20.3 (195.0)	n.s
Lymphocytes at diagnosis (cells/mm^3^)	983.0 (734.4)	919.6 (973.1)	n.s
Monocytes at diagnosis (cells/mm^3^)	501.3 (238.2)	707.1 (473.8)	n.s
Neutrophils at diagnosis (cells/mm^3^)	8261.4 (5372.3)	12182.5 (15051.7)	n.s
Basophils at diagnosis (cells/mm^3^)	13.3 (32.3)	14.2 (16.5)	n.s
Eosinophils at diagnosis (cells/mm^3^)	112.2 (231.0)	51.5 (118.8)	0.043
Ratio N/L at admission to ICU	7.2 (9.2)	11.7 (20.1)	n.s
Ratio N/L at diagnosis	7.3 (7.6)	10.4 (13.5)	0.059

Survival time was censored at day 28. Continuous variables are expressed as median (interquartile rank). Categorical variables are expressed as *n* (% over column). N.s: not significant.
